# High-Linearity and High-Speed ROIC of Ultra-Large Array Infrared Detectors Based on Adaptive Compensation and Enhancement

**DOI:** 10.3390/s23125667

**Published:** 2023-06-17

**Authors:** Zhongjie Guo, Bin Wang, Suiyang Liu, Ruiming Xu, Ningmei Yu

**Affiliations:** Department of Electronic Engineering, Xi’an University of Technology, No. 5, Jinhua South Road, Xi’an 710054, China; binwang@stu.xaut.edu.cn (B.W.); syliu@stu.xaut.edu.cn (S.L.); rmxu@stu.xaut.edu.cn (R.X.); yunm@xaut.edu.cn (N.Y.)

**Keywords:** IR ROIC, CDS, high linearity, high speed

## Abstract

In order to solve the problem of limited linearity and frame rate in the large array infrared (IR) readout integrated circuit (ROIC), a high-linearity and high-speed readout method based on adaptive offset compensation and alternating current (AC) enhancement is proposed in this paper. The efficient correlated double sampling (CDS) method in pixels is used to optimize the noise characteristics of the ROIC and output CDS voltage to the column bus. An AC enhancement method is proposed to quickly establish the column bus signal, and an adaptive offset compensation method is used at the column bus terminal to eliminate the nonlinearity caused by the pixel source follower (SF). Based on the 55 nm process, the proposed method is comprehensively verified in an 8192 × 8192 IR ROIC. The results show that, compared with the traditional readout circuit, the output swing is increased from 2 V to 3.3 V, and the full well capacity is increased from 4.3 Me- to 6 Me-. The row time of the ROIC is reduced from 20 µs to 2 µs, and the linearity is improved from 96.9% to 99.98%. The overall power consumption of the chip is 1.6 W, and the single-column power consumption of the readout optimization circuit is 33 μW in the accelerated readout mode and 16.5 μW in the nonlinear correction mode.

## 1. Introduction

Infrared detectors are widely used in military and civilian fields, whether it is for military infrared guidance, military satellite and infrared early warning system, or civilian field monitoring security, scenic spot cultural relic protection, forest fire prevention or other applications. With the improvement of imaging quality and spatial resolution requirements in military and civilian applications, infrared image sensors are developing towards a larger array, smaller pixels and higher performance. However, the ROIC of an ultra-large array infrared detector, often due to the strict restrictions on the pixel area, the parasitic effect caused by an ultra-long column bus, and the deterioration of device parameters at low temperatures, brings serious challenges to the circuit design, which makes the ROIC at ultra-high resolution have poor linearity and low frame rate.

In recent years, there have been many studies on accelerated readout and high-linearity readout schemes in infrared and visible image sensors, but all of them have certain limitations. In references [1,2], an OTA (operational transconductance amplifier) is formed by the SF in pixels and the SF on the column together and connected to the buffer. Although this method can achieve 99.92% linearity in theory, once the pixel array is large, the parasitic problem caused by the column bus will lead to the failure of the OTA, and the structure introduces an additional column bus. In reference [3], a diode-connected N-channel metal oxide semiconductor (NMOS) was used to boost the gate-source voltage lost by the NMOS SF, but the actual correction effect was limited due to the asymmetric structure and the introduction of one more column bus in each column. The scheme of generating nonlinear ramps to eliminate pixel output nonlinearity mentioned in references [4,5] can achieve 99.95% linearity, but it requires a high-precision analog-to-digital converter (ADC) and digital-to-analog converter (DAC), which introduces a large chip area and power consumption. Reference [6] has studied the influence of column bus parasitical on readout speed, but there is no effective solution. Reference [7] proposed a column-sharing acceleration structure, but this structure added an additional column bus to transmit the sampling current to the signal processing end, and the additional column bus itself also had the problem of slow settling time, which made the acceleration effect little. Although the accelerated readout circuit proposed in our previous study achieves a good acceleration effect [8], the reset signal of each readout is the fixed reset signal on the Dummy pixel, which leads to a lot of fixed-pattern noise (FPN) in the pixel that cannot be eliminated well.

In this paper, a novel analog readout circuit is proposed to solve the problems of low linearity and low frame rate in the ultra-large array IR ROIC and to solve the problems of the previous research scheme, which includes a built-in correlated Double Sampling direct injection (CDSDI) pixel and column-level readout optimization circuit. The CDS realized by the traditional direct injection (DI) pixel is a fake CDS because the integration signal and the reset signal are not in the same frame. The CDSDI pixel proposed in this paper can realize true CDS. By using the column-level optimized readout circuit outside the array, the problems of low linearity of DI pixels and longer setup times caused by column bus parasitic capacitance and resistance in large arrays are solved.

## 2. Analysis of IR ROIC Architecture

**Figure 1:**
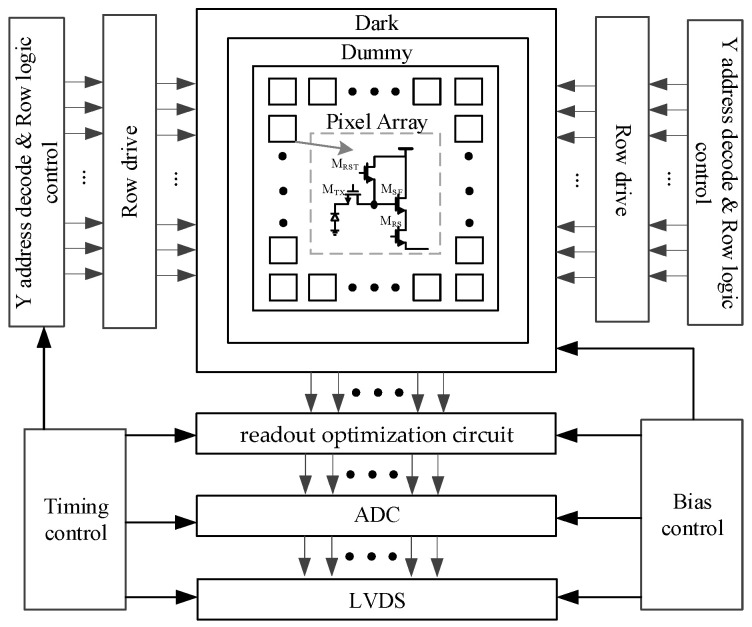
Infrared readout circuit architecture.

The IR ROIC is a mixed-signal circuit system. The overall architecture of the IR ROIC is shown in Figure 1. The analog module mainly includes a pixel array, readout optimization circuit, ADC, bias circuit, phase locking loop (PLL), etc. The digital control circuit includes address decoding, line logic control, line drive, etc. The analog module should not only ensure the quality of signal processing but also control the power consumption of the whole circuit. The digital control circuit includes address decoding, row logic control, row drive, etc., which provides reasonable timing control and signal control for the ROIC and realizes the cooperative work of each module of the ROIC.

The signal output from the pixel is transmitted to the ADC and converted into a digital signal, which is then output by the digital module. The linearity of the pixel determines the upper limit of the linearity of the whole ROIC. In the case of a large array, the conversion time of the ADC can reach 400 ns [9], and the establishment time of the bus signal under a large area array is usually at the microsecond level. Thus, the readout rate of the pixel output to the column bus signal determines the frame rate of the whole chip. 

The readout circuit designed in this paper needs to work at 110 Kelvins, and the threshold voltage VTH of the MOSFET will rise by 40%. Under the constraints of large arrays and small pixels, the large array IR ROIC often uses the DI pixel of NMOS to obtain a higher fill factor. At low temperatures, the increase in threshold voltage makes the linearity of NMOS SF worse, which leads to serious nonlinearity of the pixel output and direct current (DC) voltage loss. Moreover, the traditional DI pixel structure makes it difficult to complete the true correlation double sampling (the reset signal and the integral signal are not in the same frame) under global exposure, and it cannot eliminate the random reset noise well.

**Figure 2:**
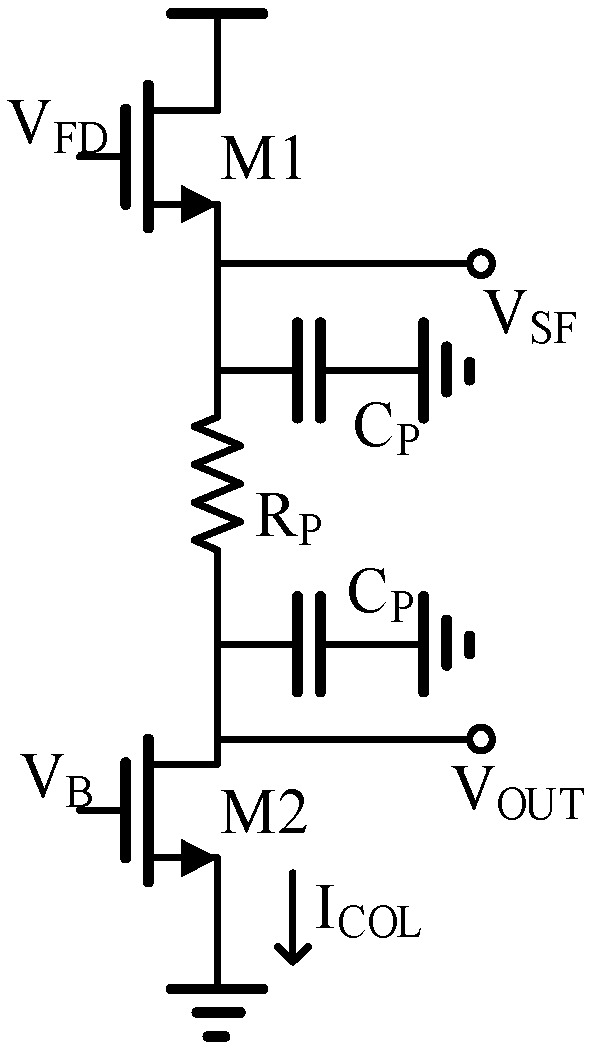
The column bus parasitic model.

The readout speed and frame rate of a super-large array IR ROIC are mainly limited by the large parasitic capacitance resistance on the array [10]. Figure 2 shows the schematic diagram of column bus parasition. The line length under the scale of the 8192 × 8192 array reaches nearly 100 mm, which makes the rise and fall time of the column bus signal very long.

## 3. Analysis of the Non-Ideal Effect of IR ROIC

### 3.1. Limitations of NMOS Type DI Pixel Structure

The DI pixel circuit commonly used in IR ROIC can realize integration while read (IWR) and integration then reading (ITR) readout mode compatibility under global exposure, but it realizes pseudo CDS under global exposure, and the signal correlation is poor, so it cannot eliminate random reset noise well. 

In practical application, the NMOS SF changes with the gate-source voltage V_GS_, and its output V_OUT_ cannot maintain the linear following effect. Considering the substrate bias effect, the small-signal gain is $A_{V}=g_{m}/(g_{m}+g_{mb})$.

(1) $$V_{TH}=V_{TH0}+\gamma \left(\sqrt{\left|2\Phi_{F}+V_{SB}\right|}-\sqrt{\left|2\Phi_{F}\right|}\right)$$

(2) $$V_{OUT}=V_{FD}-V_{TH}-V_{OD}$$

In Equation (1), *V_SB_* is the voltage between the source and substrates of NMOS SF, *V_OD_* is the overdrive voltage of NMOS SF, *γ* is the bulk effect coefficient, *V_TH_*_0_ is the threshold voltage of NMOS when *V_SB_* = 0, and Φ*_F_* is process dependent. According to the above equation, the *V_TH_* of the NMOS SF is related to *V_SB_*, and *V_SB_* = *V_OUT_*, in which case the gate-source voltage *V_GS_* of the SF is no longer constant.

For different input signals, the output voltage difference Δ*V_OUT_* of the SF is shown in Formula (3).

(3) $$\begin{array}{l} \Delta V_{OUT}=V_{OUT1}-V_{OUT2}=\Delta V_{FD}-\Delta V_{TH}=\Delta V_{FD}-\\ \gamma \left(\sqrt{\left|2\Phi_{F}+V_{OUT1}\right|}-\sqrt{\left|2\Phi_{F}+V_{OUT2}\right|}\right) \end{array}$$

where Δ*V_FD_* is the difference between the two input voltages of SF. It can be seen from Equation (3) that the nonlinear part appears after being read out by the SF as follows:

(4) $$\gamma \left(\sqrt{\left|2\Phi_{F}+V_{OUT1}\right|}-\sqrt{\left|2\Phi_{F}+V_{OUT2}\right|}\right)$$

Moreover, as the readout voltage increases, the influence of the nonlinear part increases, which seriously affects the linearity of the ROIC and reduces the imaging quality of the infrared detector. Especially at 110 K low temperature, for the process adopted in this design, the threshold voltage of 3.3 Vnmos is 300 mV higher than that at normal temperature, which leads to a more serious nonlinear response caused by substrate bias effect.

### 3.2. Effect of Parasitic Capacitance Resistance on Output

The IR ROIC of the 8192 × 8192 array has large parasitic resistance *R_P_* and parasitic capacitance *C_P_* (Figure 1).

Parasitic resistance *R_P_* mainly brings the DC voltage loss. Under the fixed column bias current, it can be concluded from Equation (6) that the voltage loss is fixed.

(5) $$V_{OUT}=V_{FD}-V_{GS1}-I_{COL}\times R_{P}$$

(6) $$\Delta V_{OUT}=V_{OUT2}-V_{OUT1}=V_{SF2}-V_{SF1}$$

Therefore, the signal establishment time caused by the parasitic capacitor *C_P_* is mainly considered. When CDS voltage is output in the column bus, the SF charges the parasitic capacitor, and the charging time can be expressed as *T_C_*, where *I_C_* is the total charging current on the column, *V_OD_*_1_ is the overdrive voltage of the SF M1, and *V_DS*2*min_* is the minimum overdrive voltage of the current mirror. *C_PL_* is the total parasitic capacitance on the column bus; *K*_1_ is the gain factor of M1, and it can be expressed as K1 = μ_n_C_ox_(W/L)_1_; μ_n_ is electronic mobility, C_ox_ is the grid oxide capacitance per unit area, and (W/L)_1_ is the aspect ratio of M1. The charging time *T_C_* is expressed as follows:

(7) $$T_{C}=\frac{C_{PL}\Delta V}{I_{C}}=\frac{C_{PL}\left(V_{OUT}-V_{DS2min}\right)}{K_{1}V_{{}_{OD1}}^{2}-I_{COL}}$$

As the output voltage increases, the charging current *I_C_* decreases, and the signal rising speed decreases. When the column bus outputs CDS voltage, the parasitic capacitor discharges through the column bias current, and the discharge time *T_D_* is expressed as follows:

(8) $$T_{D}=\frac{C_{PL}\left(V_{OUT}-V_{DS2min}\right)}{I_{COL}}$$

To speed up the reading speed, the most direct way is to increase the column bus current, but this will introduce a huge power consumption, so the reasonable solution is to provide an additional dynamic current for the column line to charge and discharge.

## 4. High Linear High Frame Rate Readout Method for Very Large Array IR Detector

In order to solve the defects of the traditional DI pixel circuit and the problem of signal establishment caused by super-large parasitization in the current super-large array IR ROIC, the new pixel proposed in this paper outputs the CDS voltage to the column bus, which shortens the row time compared with the output of two voltage signals from the traditional pixel. Then the column bus voltage signal is accelerated and nonlinearly corrected by the column readout optimization circuit. Finally, the design index of high frame rate and high linearity is achieved.

### 4.1. CDSDI Pixel Circuit Design

**Figure 3:**
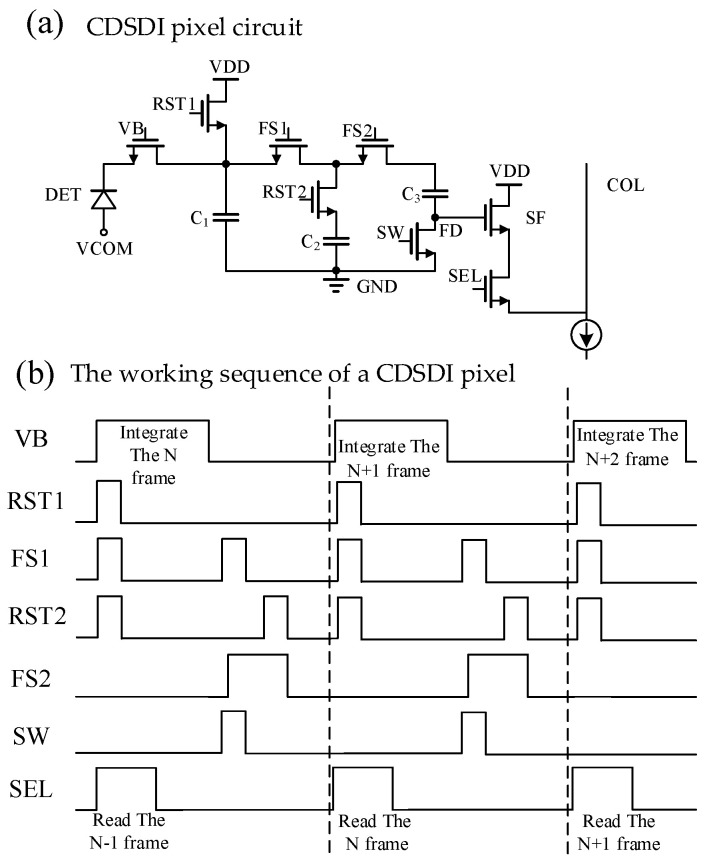
CDSDI pixel and Working time sequence.

The CDSDI pixel proposed in this paper is shown in Figure 3a. DET is the mercury cadmium telluride (MCT) infrared diode. C_1_, C_2_ and C_3_ are sampling capacitors; NMOS VB and VCOM jointly determine the bias voltage of DET. C_2_ stores the reset voltage VRST of the current frame, and C_3_ stores the signal voltage VSIG of the current frame. The voltage of the C_2_ bottom plane is raised to VRST through the C_3_ upper plate so that the CDS voltage of the current frame is stored in the C_2_ bottom plane and the reset voltage is stored in C_2_ when the next frame is reset, so as to realize the true CDS function in the DI pixel circuit under global exposure. At the same time, in order to ensure that the CDS voltage can still be read normally when the CDS voltage is very low, the power supply voltage of the pixel is designed to be 4.3 V and the GND voltage is designed to be 1 V, so that the final output voltage of the pixel reaches 3.3 V. The pixel array is independently powered, so it is easier to implement.

The specific working sequence of a CDSDI pixel is shown in Figure 3b. Firstly, the global reset operation is carried out. At this time, VB, RST1, RST2, FS1 and FS2 are on, and the reset voltage VRST is stored in C_1_ and C_2_. Then, the global integration operation is conducted, followed by VB conduction and C_1_ storage signal voltage VSIG. Then, charge transfer occurs, followed by FS1, SW and FS2 conduction, and C_1_ charge transfer to C_3_; then RST2, FS2 conduction and other switches are disconnected, the CDS within the pixel, the C_3_ upper plate voltage is VRST, the C_3_ bottom plate voltage is VRST-VSIG. Finally, SEL is conducted, and the CDS voltage of the C_3_ bottom plate is read out row by row through the SF to the column line. 

The pixel size of this design is 10 μm × 10 μm, C_1_ and C_2_ are NMOS capacitance, where C_1_ is 296 fF, and the calculation formula of full well capacity is as follows, so the full well capacity of this design pixel is 6 Me-.

(9) $$N_{fullwell}=\frac{C_{1}V_{outswing}}{q}$$

Compared with the traditional DI pixel structure in reference [11], the swing amplitude is increased from 2 V to 3.3 V, and the full well capacity is increased from 4.3 Me- to 6 Me-. Moreover, the CDS function is integrated into the pixel, which, on the one hand, eliminates the CDS circuit at a later stage and reduces the area and power consumption. On the other hand, compared with the traditional pixel structure, the two voltage signals of readback and integration are read out. The pixel circuit proposed in this paper outputs a CDS voltage signal, which can speed up the readout rate by one time. 

### 4.2. Column-Level Readout Optimization Circuit Based on Adaptive Offset Compensation and AC Enhancement

Because the nonlinearity and slow signal establishment in the super-large array infrared readout circuit seriously affect the performance of the detector, this paper proposes a column-level readout optimization circuit that can realize nonlinear correction and accelerated readout. In the accelerated readout mode, the AC enhancement circuit is formed by the amp to accelerate the establishment of the column bus signal to realize the accelerated readout function. After the signal of the column bus is established, the readout optimization circuit works in the nonlinear correction mode, and the nonlinear correction function is realized by the adaptive offset compensation of the SF through the negative feedback loop. 

**Figure 4:**
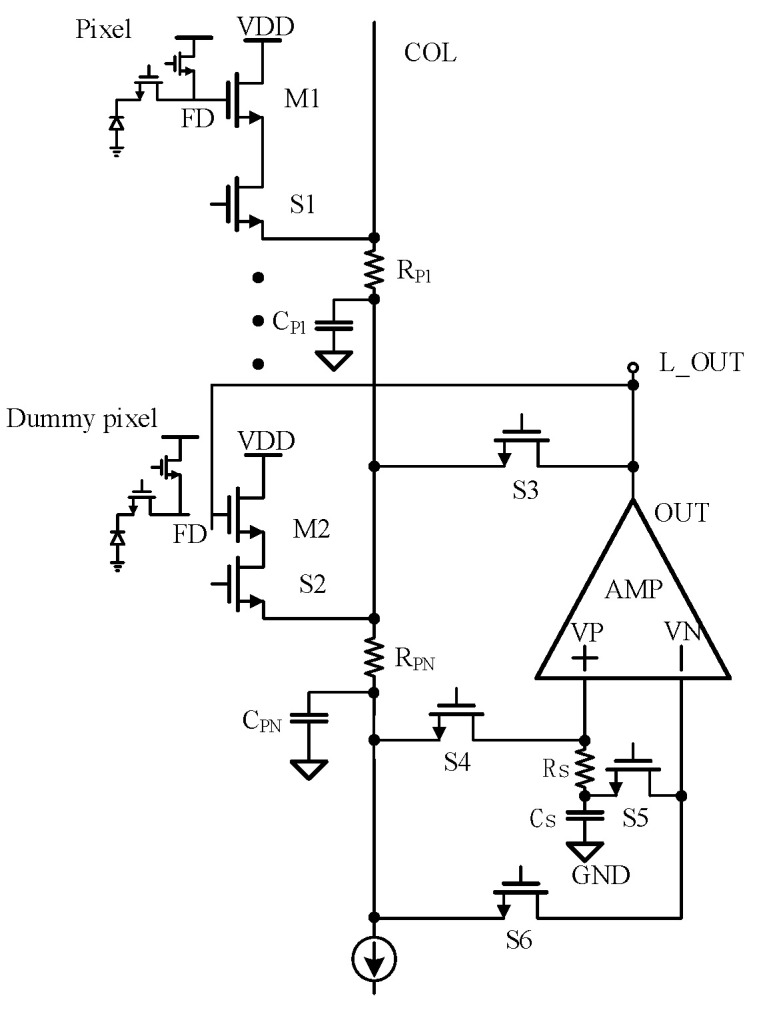
Column-level readout optimization circuit structure.

Figure 4 shows the column-level readout optimization circuit proposed in this paper. The working mode of the circuit is controlled by NMOS switches S1 to S6. When switches S1, S3, S4 and S5 are on, the circuit is in accelerated readout mode. When switches S2 and S6 are on, the circuit is in nonlinear correction mode.

The OTA in the circuit adopts the rail-to-rail structure applied at low temperatures mentioned before [12] and the slew rate enhancement circuit in reference [13]. When the circuit is in accelerated readout mode, the slew rate enhancement structure is turned on to speed up the signal establishment, and when the circuit is in nonlinear correction mode, the slew rate enhancement structure is turned off to reduce power consumption.

The specific principle of accelerated readout mode is that when there is a voltage difference between the VP and VN of the OTA, a large dynamic output current is generated to charge and discharge the column bus. When S1 is turned on to read the signal of the selected pixel, the voltage of the FD node of the photosensitive pixel will be read to the column line through the SF M1. However, the signal establishment is very slow due to the existence of parasitic capacitance *C_P_* to the ground on the long column line. At this time, the charging current of the column bus will also charge the sampling capacitor *C_S_*. The charging current of the column bus will generate a voltage difference through the resistance *R_S_*, and the VP voltage of the OTA is greater than VN, so the OTA will output a large current to the column bus to achieve the effect of readout acceleration. At this time, the output current of OTA is *I_O_*, where *A_i_* is the OTA gain and *I_s_* is the current flowing through the resistance *R_S_*.

(10) $$I_{O}=I_{S}\times A_{i}=\frac{sC_{S}V_{OUT}}{1+sR_{S}C_{S}}\times A_{i}$$

In combination with Equation (7), the charging time of the column bus is as follows:

(11) $$T_{C}=\frac{C_{P}\left(V_{OUT}-V_{DS2MIN}\right)}{K_{n1}V_{OD1}^{2}+I_{O}-I_{COL}}$$

When the voltage on *C_S_* is equal to the output voltage of the column bus, no more current will flow through the resistance *R_s_*. At this time, the VP voltage is equal to VN, and the acceleration ends.

In the discharge phase, the voltage of VN is greater than the VP. At this time, the OTA output a negative current and the parasitic capacitor *C_P_* and the sampling capacitor *C_S_* are discharged. When the voltage in the capacitor drops to the lowest working voltage *V_OD_* of the current source of the line, the voltage of the VN and VP of OTA is equal. The discharge time of the capacitor can be expressed as follows:

(12) $$T_{C}=\frac{C_{P}\left(V_{OUT}-V_{DS2MIN}\right)}{I_{O}+I_{COL}}$$

As can be seen from the above two equations, OTA outputs a large dynamic charge and discharge current I_O_ to charge the column parasitic current, which can greatly reduce the rise and fall time of the column bus signal.

The column acceleration circuit [8] we proposed before only accelerates the integrated signal of the photosensitive pixel, and the reset signal read out is from the Dummy pixel. Therefore, the double sampling voltage is not correlated. The ROIC proposed in this paper completes CDS in pixels and only needs to accelerate the readout of CDS voltage, which solves the previous problem.

Because of the parasitic capacitance resistance caused by the ultra-long column bus, the signal establishment time of the column bus is about 10 μs without acceleration optimization, and the signal establishment time of the column bus is less than 1 μs after acceleration optimization.

When the circuit works in nonlinear correction mode, it is mainly composed of NMOS switches S4 and S5, sampling capacitor C_S_ and OTA.

The whole nonlinear correction structure is constructed by connecting the SF output in the photosensitive pixel with the SF in the Dummy pixel. Firstly, switches S4 on and S5 off and the pixel output signal is read to the capacitor C_S_. Then, S4 is turned off and S5 is on. The VN and VP voltages of OTA are equal, so the substrate bias of the Dummy SF is the same as that of the photosensitive pixel SF, the bias current is the same column bus current, the width-length ratio is the same, and S1 and S2 are the same NMOS. Therefore, the voltage on the capacitor *C_S_* is lifted to VGS by the Dummy SF. As the width-length ratio of the two SF is the same and the layout is the same, the VGS are the same, so the voltage on the capacitor *C_S_* is restored to the FD point voltage by the Dummy SF, and the adaptive compensation for the contrast bias effect of the pixel SF is realized.

Equations (13) and (14) are the expressions when pixels output different CDS voltages (*V_FD_*_1_, *V_FD_*_2_) according to a constant column bias current.

(13) $$K_{1}{\left(V_{G1,FD1}-V_{S1,FD1}-V_{TH1,FD1}\right)}^{2}=K_{1}{\left(V_{G1,FD2}-V_{S1,FD2}-V_{TH1,FD2}\right)}^{2}$$

(14) $$K_{2}{\left(V_{G2,FD1}-V_{S2,FD1}-V_{TH2,FD1}\right)}^{2}=K_{2}{\left(V_{G2,FD2}-V_{S2,FD2}-V_{TH2,FD2}\right)}^{2}$$

Δ*V_FD_* is the voltage difference between two CDS voltages output by the FD point pixel, and Δ*V_OUT_* is the voltage difference between two CDS voltages output by a linearized circuit. The calculation results are shown in Equations (15) and (16).

(15) $$\Delta V_{FD}=V_{G1,FD1}-V_{G1,FD2}=V_{S1,FD1}-V_{S1,FD2}-\left(V_{TH1,FD1}-V_{TH1,FD2}\right)$$

(16) $$\Delta V_{OUT}=V_{G2,FD1}-V_{G2,FD2}=V_{S2,FD1}-V_{S2,FD2}-\left(V_{TH2,FD1}-V_{TH2,FD2}\right)$$

Considering that there is an offset voltage *V_OS_* in the OTA, there is always a deviation V_OS_ in the source voltage of M1 and M2. After the nonlinear correction circuit, the error voltage of the output is shown in Equation (17).

(17) $$\Delta V_{FD}-\Delta V_{OUT}=\gamma \left(\begin{array}{l} \sqrt{\left|2\Phi_{F}+V_{S1,FD1}\right|}-\\ \sqrt{\left|2\Phi_{F}+V_{S1,FD2}\right|} \end{array}\right)-\gamma \left(\begin{array}{l} \sqrt{\left|2\Phi_{F}+V_{S1,FD1}-V_{OS}\right|}-\\ \sqrt{\left|2\Phi_{F}+V_{S1,FD2}-V_{OS}\right|} \end{array}\right)\;$$

At this time, the nonlinear part becomes the subtraction of two very similar values in the formula, which greatly reduces the nonlinear influence.

The current matching between different columns is poor in the large-array ROIC. In our previous study, we used SF, OTA and mirror SF outside the array to form a negative feedback loop to raise the output voltage to VGS of the mirror SF [14]. One of the problems caused by this structure is that each column introduces an extra current. Second, considering the mismatch between SF in the pixel array and SF outside the array, as well as the mismatch of the current source, the correction effect will be greatly reduced. In this paper, we propose that the structure uses SF in Dummy to form a feedback loop, which has a better match with SF in the photosensitive pixel and uses time-sharing column bus current to improve the nonlinear correction effect and reduce power consumption.

## 5. Verification Results and Analysis

**Figure 5:**
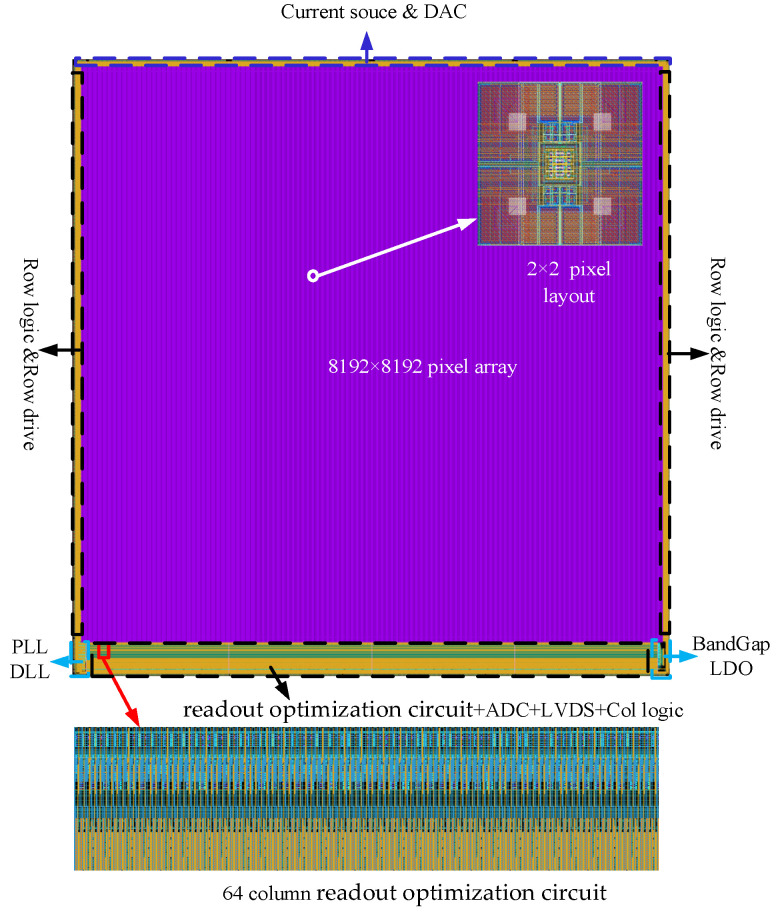
8192 × 8192 ROIC layout.

All the research results presented in this paper were verified and analyzed by the 55 nm process. Figure 5 shows the overall layout of the 8192 × 8192 large array IR ROIC. The pixel layout is designed with 2 × 2 as the minimum unit to make more efficient use of the layout area and adaptive stitch process. The pixel array is surrounded by 32-turn Dark and Dummy pixels, and the column-level readout optimization circuit is placed below the pixel array. Overall, the 8191 × 8192 IR ROIC chip size is 100 mm × 110 mm, and the 8192 column readout optimization circuit size is 8192 × 100 µm × 10 μm, the area only accounts for 0.082% of the whole.

**Figure 6:**
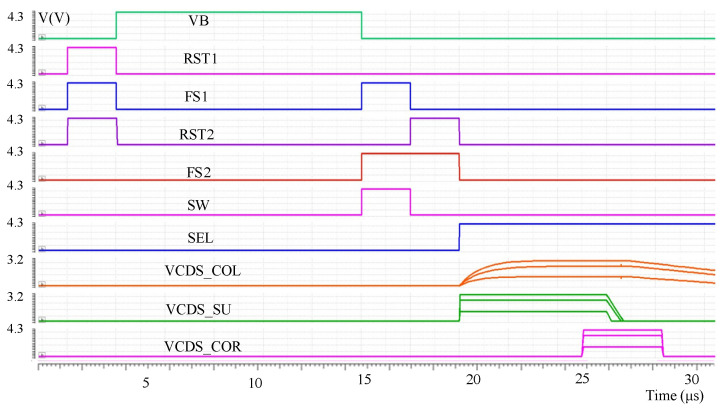
Pixel and readout optimization circuit simulation results.

The proposed circuit was verified by simulation based on the 8192 × 8192 IR ROIC layout. The overall performance of the proposed circuit structure is verified by comparing the output results of the pixel and open readout optimization circuits under different integrating currents through simulation. The overall readout sequence is as follows: first, when the pixel starts to output the CDS voltage signal VCDS_COL, the readout optimization circuit is in the accelerated establishment mode to accelerate the establishment of the pixel output VCDS_COL and output the accelerated VCDS_SU signal; then the readout optimization circuit is in the nonlinear correction mode, and VCDS_COR is output after the linearization of the VCDS_SU signal. Figure 6 shows the simulation results of circuits with different integral current (CDS voltage from high to low; integral current is 100 nA, 80 nA, 50 nA), where VCDS_COL is the CDS voltage directly output by pixel to the column bus and VCDS_SU is the voltage established by readout optimization circuit acceleration. VCDS_COR is the output of the signal with nonlinear correction after accelerated establishment.

It can be seen from the simulation results that the readout optimization circuit makes the signal rise and fall more than ten times faster, and the output of the pixel circuit can reach up to 3 V. After nonlinear correction, the nonlinear error caused by the SF can be compensated, and the swing amplitude can also reach 3.3 V while improving the linearity.

**Figure 7:**
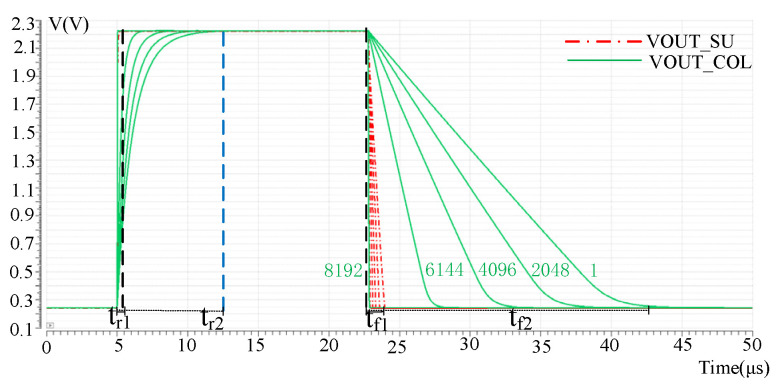
Different row pixel output after the readout acceleration effect.

In the process of reading out pixel array signals, the effective length of the column bus will change when different rows are read. The first row of pixels has the longest effective column bus length and the largest parasitic capacitor and resistance, so the signal needs the longest time to establish. The 8192 row has the least effective column bus length. So the parasitic parameter is the smallest, and the signal is established the fastest. Therefore, the signal establishment time of the pixel array decreases from top to bottom. Therefore, the establishment of VCDS output by pixels in row 1, row 2048, row 4096 and row 8192 is verified by simulation, respectively, as shown in Figure 7. Where VOUT_COL and VOUT_SU are the read waveforms of the pixel output signal on the column line without column acceleration and after column acceleration. It can be seen that the pixel in the first row has the largest parasitic effect and the acceleration effect is the most obvious. The rise and fall time of the signal after the acceleration of the pixel in the first row is t_r1_ and t_f1_, respectively, and the rise and fall time before the acceleration of the pixel in the first row is t_r2_ and t_f2,_ respectively. The values of t_r1_ and t_f1_ were 493 ns and 994 ns, respectively, while the values of t_r2_ and t_f2_ without acceleration were 7 μs and 15.7 μs, respectively.

Therefore, the readout optimization circuit proposed in this paper greatly speeds up the readout of the column bus signal. The power consumption in the accelerated readout mode is 10 μA, accounting for 0.2% of the entire chip power consumption, and the row time is less than 2 μs. The high frame frequency of the 8192 × 8192 infrared readout circuit is achieved, and the technical problem of the insufficient frame frequency of the ultra-large array infrared readout circuit is solved.

**Figure 8:**
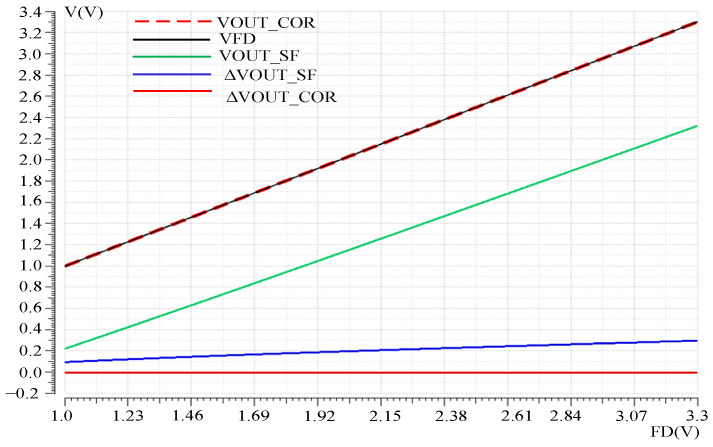
Voltage following before and after nonlinear correction.

The nonlinear correction effect of the readout optimization circuit was verified by simulating the DC response curve of the pixel SF output VOUT_SF and the output VOUT_COR of the nonlinear correction mode of the readout optimization circuit. The variable was pixel FD voltage VFD. The simulation results are shown in Figure 8, where ΔVOUT_SF is the difference between the pixel SF output and FD point voltage, and ΔVOUT_COR is the difference between the nonlinear correction output and FD voltage. It can be seen that when VOUT_SF changes in the VFD, the error voltage changes nonlinearly with the change in the VFD voltage, reaching a maximum of 300 mV. Reduce the output swing and linearity of the readout circuit. The output VOUT_COR follows the VFD well, with a maximum error voltage of 300 μV, reducing the nonlinear error voltage by nearly a thousandfold and increasing the output swing by 300 mV.

The linearity of IR ROIC can be expressed by the following formula, where Δ*V_max_* is the maximum voltage deviation and *V_OUT_*__*SWING*_ is the full swing voltage.

(18) $$Linearity=1-\frac{\Delta V_{\max}}{V_{OUT\_SWING}}$$

**Figure 9:**
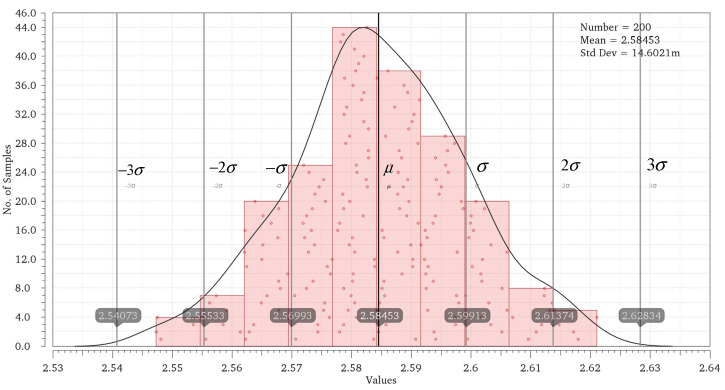
MC simulation.

**Figure 10:**
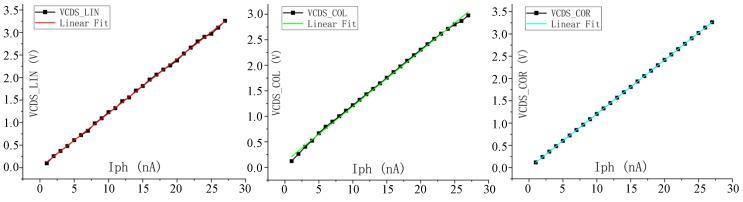
Output fits the curve.

Through simulation verification of the proposed pixel circuit and combined readout optimization circuit, the output current of the equivalent model of the infrared detector is set to be 100 pA~30 nA and the integration time is 32 μs. At the same time, Monte Carlo (MC) simulation is carried out considering the mismatch of two current sources, M7 and M10, of the linearized circuit [14] proposed before. The maximum CDS voltage and minimum CDS voltage obtained by MC simulation under different integrating currents are fitted to the curve to get the worst possible result after tape out, and Figure 9 shows the MC simulation results under 23 nA integrated current. VOUT_COL is the voltage directly output by the pixel, VOUT_COR is the output after the nonlinear correction of the readout optimized circuit, and VOUT_LIN is the output of the linearized circuit we proposed before. The simulation data and fitting curves of the three outputs are shown in Figure 10. The maximum error voltage of VOUT_COL data and fitting data is 0.093 V, and the output swing of the pixel is 3 V. In this case, the nonlinear error of the readout circuit is 3.1%, and the linearity is 96.9%. The linearity of output VOUT_COR is 99.98%, and the linearity of output VOUT_LIN is 98.86%.

It can be seen that the structure proposed in reference [13] will greatly reduce the nonlinear correction effect when considering the influence of device mismatch, especially the mismatch of two current sources. The structure proposed in this paper does not reduce the correction effect due to the dummy SF multiplexing of the same column current. Compared with the previous structure, not only the linearity is improved, but also the current of mirror SF is reduced. The single-column readout optimization circuit introduces only 5 μA power consumption in the nonlinear correction mode, accounting for less than 0.1% of the total power consumption of the chip.

**Figure 11:**
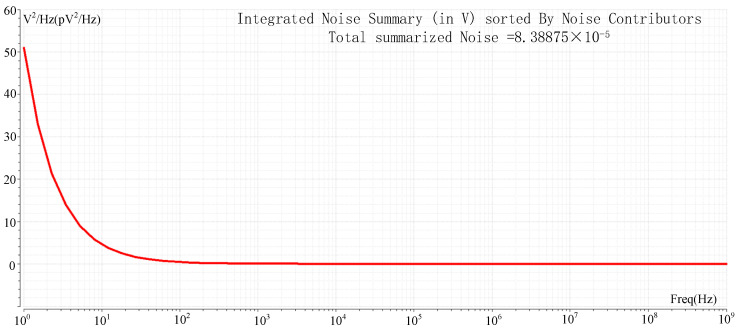
Noise simulation.

Noise in ROIC mainly includes spatial domain noise and time domain noise, and spatial domain noise can be well eliminated by CDS, while time domain noise is mainly suppressed by device parameter optimization and time sequence improvement. The readout optimization circuit proposed in this paper is mainly based on DC voltage signals under the column bus acceleration mode, so the noise introduced in the nonlinear correction process is mainly considered. Figure 11 shows the simulation results for the noise of ROIC. The integrated noise of 1 Hz~1 GHz is 83.8 μV. The noise is converted to 33 e^−^ at the pixel FD node. The dynamic range (DR) is 85.1 dB, and no significant noise is introduced compared with other ROIC or linearity-enhancing circuits. For a detailed comparison with other literature, see Table 1.

Table 1 shows the comparison between the linearity of the ROIC designed in this paper and references [1,2,3,4,5]. By comprehensive comparison of full well capacity, pixel size and DR, this design still achieves a good design index. It can be seen that the design method proposed in this paper not only guarantees linearity but also controls power consumption well and realize high DR.

Table 2 shows the comparison between the frame rate of the readout circuit designed in this paper and references [7,15,16,17]. It can be seen that the design method proposed in this paper can effectively improve the frame rate of the ultra-large array infrared readout circuit.

## 6. Conclusions

This paper presents a design method of high frame rate and high linearity for the 8192 × 8192 array IR ROIC, including a CDSDI pixel and column-level readout optimization circuit, through the column bus feedback and time-division multiplexing of column bus current to achieve accelerated readout and nonlinear correction of pixel output. With a size of 10 μm pixel center distance on a chip with an area of 100 mm × 110 mm, the pixel with CDS function is realized on the chip with 3.3 V output swing and 6 Me- full well capacity. The row time is reduced to 2 μs, the frame rate is 80 frames, and the linearity is 99.98%. The power consumption of the readout-optimized circuit is 10 μA at acceleration mode and 5 μA at nonlinear correction mode. Compared with the existing research results, the ROIC we proposed has the highest linearity and the fastest readout speed, and no additional column bus is introduced. The readout optimization circuit is distributed on the periphery of the pixel array. At the same time, compared with the readout auxiliary circuit proposed in other references, the power consumption is lower, the effect is better, and the circuit area is smaller. Moreover, the ROIC design scheme proposed in this paper can be applied to other large array sensor readout circuits and provides a practical and effective solution for the design of high-linearity and high-speed large array IR ROIC.

## Figures and Tables

**Table 1:** Linearity compared with other references.

Reference	[1]	[2]	[3]	[4]	[5]	This Work
Column consumption	126 μW	68 μW	-	96.3 μW	-	16.5 μW
linearity	99.92%	99.7%	99.89%	99.95%	99.97%	99.98%
Pixel size (μm)	7 × 7	7 × 7	15 × 15	6.5 × 6.5	12 × 10	10 × 10
Full well capacity (e^−^)	-	-	7 M	91.7 K	23.1 K	6 M
Read noise	411 nV^2^	5.05 μV^2^	-	7.9 e^−^	3.6 e^−^	33 e^−^/83.3 μV
DR (dB)	-	-	81	81.3	76.0	85.1
additional column bus	1	1	1	0	0	0

**Table 2:** Frame rate is compared with other references.

Reference	[7]	[15]	[16]	[17]	This Work
Array	128 × 3	26,112 × 15,000	15,360 × 15,360	2304 × 2304	8192 × 8192
Pixel size (μm)	15	3.9	7.5	55	10
Process	0.35 μm	65 nm	55 nm	0.18 μm	55 nm
Chip size (mm)	3.84 × 2.58	105.2 × 65.63	120 × 120	125 × 125	100 × 110
Row time (μs)	0.008	6.6	6.5	36.2	1.5
Rate	1000	1	10	12	80

## Data Availability

The data presented in this study are available on request from the corresponding author. The data are not publicly available due to privacy.

## References

[1] Teymouri M., Sobhi J. (2018). An ultra-linear CMOS image sensor for a high-accuracy imaging system. Int. J. Circuit Theory Appl..

[2] Teymouri M. (2019). A highly linear and high-accurate CMOS image sensor. Analog. Integr. Circuits Signal Process..

[3] Niu Y.Z., Zhu Y.J., Lu W.G., Gu Y.T., Zhang Y.C., Chen Z.J. (2020). A readout structure with double column buses and shared source follower for IRFPAs. J. Infrared Millim. Waves.

[4] Li C., Han B., He J., Guo Z., Wu L. (2020). A Highly Linear CMOS Image Sensor Design Based on an Adaptive Nonlinear Ramp Generator and Fully Differential Pipeline Sampling Quantization with a Double Auto-Zeroing Technique. Sensors.

[5] Wang F., Theuwissen A.J.P. (2018). Pixel Optimizations and Digital Calibration Methods of a CMOS Image Sensor Targeting High Linearity. IEEE Trans. Circuits Syst. I Regul. Pap..

[6] Gao J., Zhang D., Nie K., Xu J. (2019). Analysis and Optimization design of the column bus parasitic effects on large-array CMOS image sensor. Microelectron. J..

[7] George S.S., Bocko M.F., Ignjatovic Z. (2015). Current Sensing Assisted Active Pixel Sensor for High-Speed CMOS Image Sensors. IEEE Sens. J..

[8] Guo Z., Cheng X., Xu R., Su C., Li C., Wang B., Guo Y., Wang Y. (2023). A 1Gpixel 10FPS CMOS image sensor using pixel array high-speed readout technology. Integration.

[9] Guo Z., Wang Y., Xu R., Yu N. (2023). High-Speed Fully Differential Two-Step ADC Design Method for CMOS Image Sensor. Sensors.

[10] Xu J., Li W., Nie K., Han L., Zhao X. (2018). A Method to Reduce the Effect on Image Quality Caused by Resistance of Column Bus. IEEE Trans. Very Large Scale Integr. Syst..

[11] Wu S.J., Yao L.B., Li D.S., Ji Y.L., Yang C.L., Li H.F., Luo M., Li M., Xu R.H. (2021). Small Pixel 10μm Pitch Infrared Focal Plane Array ROIC Design. Infrared Technol..

[12] Wang B., Guo Z.-J., Wang Y.-L., Guo Y.-M., Xu R.-M., Cheng X.-Q. A linearization technique for Cryogenic infrared readout circuit. Proceedings of the 2022 IEEE 16th International Conference on Solid-State & Integrated Circuit Technology (ICSICT).

[13] Ye Z.H., Yang H.G., Li F.Y., Cheng X.Y., Yin T., Liu F. (2012). A Universal High-Speed, Low-Power Dynamic Slew-Rate Enhancement Circuit for Large Capacitancer. Microelectron. Comput..

[14] Guo Z., Yu N., Wu L. (2021). An Improved Global Shutter Pixel with Extended Output Range and Linearity of Compensation for CMOS Image Sensor. Chin. J. Electron..

[15] Bogaerts J., Lafaille R., Borremans M., Guo J., Ceulemans B., Meynants G., Sarhangnejad N., Arsinte G., Statescu V., van der Groen S. 6.3 105 × 65mm2 391Mpixel CMOS Image Sensor with >78 dB Dynamic Range for Airborne Mapping Applications. Proceedings of the 2016 IEEE International Solid-State Circuits Conference (ISSCC).

[16] Guo Z., Yu N., Wu L. (2020). A synchronous driving approach based on adaptive delay phase-locked loop for stitching CMOS image sensor. IEICE Electron. Express.

[17] Kim M.S., Kim G., Cho G., Kim D. (2016). Development of a 55 μm pitch 8 inch CMOS image sensor for the high resolution NDT application. J. Instrum..

